# Association between post-diagnostic use of cholera vaccine and risk of death in prostate cancer patients

**DOI:** 10.1038/s41467-018-04814-4

**Published:** 2018-06-18

**Authors:** Jianguang Ji, Jan Sundquist, Kristina Sundquist

**Affiliations:** 1 Center for Primary Health Care Research, Lund University/Region Skåne, Malmö, 20502 Sweden; 2 Department of Family Medicine and Community Health, Department of Population Health Science and Policy, Icahn School of Medicine at Mount Sinai, New York, NY USA

## Abstract

Recent evidence suggests that cholera toxin might have multiple functions regarding the ability to regulate the immune system. However, it is unknown whether subsequent administration of cholera vaccine might affect the mortality rate in patients with prostate cancer. Here we report that patients in Sweden, who were diagnosed with prostate cancer between July 2005 and December 2014 and used cholera vaccine, have a decreased risk of death from prostate cancer (HR, 0.57; 95% CI, 0.40–0.82) as compared to patients with prostate cancer but without cholera vaccine use, adjusted for a range of confounding factors. In addition, patients using cholera vaccine show a decreased risk of death overall (HR, 0.53; 95% CI, 0.41–0.69). The decreased mortality rate is largely consistent, irrespective of patients’ age or tumor stage at diagnosis. In this population-based study, we suggest that subsequent administration of cholera vaccine after prostate cancer diagnosis might reduce the mortality rate.

## Abstract (web summary)

Post-diagnostic use of cholera vaccine can improve the prognosis in patients with colorectal cancer suggesting cholera toxin might have an antineoplastic effect. Here the authors perform a nationwide population-based study and report an association between cholera vaccine administration and reduction in prostate cancer mortality.

## Introduction

A steady decline in prostate cancer mortality has been reported during the last decade, including in the United States and in many European countries^1,2^. The mean 5-year relative survival rate in Europe is around 83%; however, the survival rate ranges from 76 to 88%^1^. Prostate cancer survival is highly dependent on the age of the patient and tumor stage at diagnosis. The survival rate is higher in patients who are diagnosed at a younger age and among those with a local or regional prostate cancer^1^. For men diagnosed with prostate cancer, which has spread to other parts of the body, the 5-year survival rate is only 30%^2^. Thus, it is highly necessary to develop new therapy options for patients with advanced prostate cancer.

Recent evidence suggests that cholera toxin, which consists of two subunits: the A subunit (CTA) and the B subunit (CTB), is a multifunctional protein with the potential ability to regulate the immune system in multiple ways^3^. CTB has adjuvant activity for mucosal vaccines; this may be due to the enhanced antigen presentation by various types of antigen-presenting cells, such as macrophages and dendritic cells^4,5^. In addition to its adjuvant properties, CTB has the function to potentially act as an anti-inflammatory agent by modulating specific signal transduction pathways and it can also act as an immunomodulatory agent to treat various autoimmune disorders^4,5^. Oral administration of cholera toxin can upregulate the accumulation of macrophages, NK cells and the regulatory T cells, as well as IL-10 production, and can downregulate the accumulation of neutrophils^3^. The immunomodulatory function of CTB might be due to its specific properties, such as the ability of binding to specific GM1 ganglioside receptors present in the gut mucosa, and facilitating antigen uptake and presentation. Previous studies have found that MAPK phosphatase-1 expression can be induced by CTB alone and can subsequently inhibit the activation of Janus kinase and p38, thus leading to a substantial attenuation of TNFα and IL-6 production from macrophages^6^. Based on the evidence presented above, we hypothesized that the immune function in prostate cancer patients might be altered when they receive cholera vaccination that includes killed whole cells of Vibrio cholera O1 and recombinant cholera toxin B subunit. Cholera vaccine might be repositioned (drug repurposing) and might be used as adjuvant therapy in patients with prostate cancer. Our previous study found that post-diagnostic use of cholera vaccine (including killed *Vibrio cholerae* O1 whole cells and recombinant CTB) can improve the prognosis in patients with colorectal cancer, which suggests that cholera toxin might have an antineoplastic effect^7–13^. In this study, we aimed to explore whether post-diagnostic use of cholera vaccine might be associated with a reduced mortality rate in patients diagnosed with prostate cancer. By retrieving data from several national Swedish registers, we could study the rates of prostate cancer mortality and overall mortality among patients who received cholera vaccine, and compare them with patients who did not receive cholera vaccination. To the best of our knowledge, this is the first nationwide population-based study to explore the association between post-diagnostic use of cholera vaccine and the risk of mortality in patients with prostate cancer.

Here, we report that patients in Sweden, who were diagnosed with prostate cancer between July 2005 and December 2014 and used cholera vaccine, have a decreased risk of death from prostate cancer (hazard ratio (HR), 0.57; 95% confidence interval (CI), 0.40–0.82), as compared to patients with prostate cancer who did not use cholera vaccine, adjusted for a range of confounding factors. In addition, patients using cholera vaccine showed a decreased risk of death overall (HR, 0.53; 95% CI, 0.41–0.69). The decreased mortality rate is largely consistent, irrespective of the age of the patient or tumor stage at diagnosis. In this population-based study, we suggest that subsequent administration of cholera vaccine after prostate cancer might reduce the mortality rate.

## Results

### Characteristics in patients with prostate cancer

From the Swedish Cancer Registry and Prescribed Drug Register, we identified 841 patients who were prescribed with cholera vaccine after their prostate cancer diagnosis between July 2005 and December 2014 (Table 1). The mean internal time from the diagnosis of prostate cancer to vaccination was 27.1 months, and 58.9 months from vaccination to the end of follow-up. The median age at diagnosis was 64 years. Compared to patients without cholera vaccination, patients who were given a cholera vaccination were diagnosed at a younger age and at an earlier stage, associated with a higher education, a higher disposable income, living in big cities, and with a lower rate of comorbidities.

**Table 1:** Baseline demographic and clinical characteristics of patients with prostate cancer, stratified by post-diagnostic use of cholera vaccine

Characteristic		Post-diagnostic users of cholera vaccine		Without cholera vaccine		*P* value
		No. of patients	%	No. of patients	%	
Overall		841	100	89,142	100	
Time interval, mean (SD)	Diagnosis to vaccine	27.1 (21.6)				
	Vaccine to end of follow-up	58.9 (26.1)				
Age at diagnosis	<60	225	26.8	12,028	13.5	<0.0001
	60–64	220	26.2	15,972	17.9	
	65–69	255	30.4	21,000	23.6	
	70+	141	16.8	40,142	45.0	
	Median age, years	64		68		
Year at diagnosis	2005–2009	628	74.8	36,884	41.4	<0.0001
	2010–2014	213	25.4	52,258	58.6	
	Median	2008		2010		
Highest education, years	1–9	154	18.3	30,041	33.7	<0.0001
	10–11	302	36.0	33,613	37.7	
	12+	385	45.8	25,488	28.6	
Birth country	Sweden	795	94.6	81,179	91.1	0.002
	European countries	38	4.5	6343	7.1	
	Others	8	1.0	1620	1.8	
Income	Lowest	71	8.5	22,870	25.7	<0.0001
	Middle-low	129	15.4	22,228	24.9	
	Middle-high	213	25.4	22,129	24.8	
	Highest	428	51.0	21,915	24.6	
Region	Big cities	450	53.6	42,184	47.3	0.001
	Southern Sweden	254	30.2	29,232	32.8	
	Northern Sweden	137	16.3	17,726	19.9	
Stage at diagnosis	Stage 1	538	64.0	43,244	48.5	<0.0001
	Stage 2	200	23.8	25,526	28.6	
	Stage 3	68	8.1	11,759	13.2	
	Stage 4	35	4.2	8613	9.7	
Chronic ischemic heart disease	No	750	89.3	73,991	83.0	<0.0001
	Yes	91	10.8	15,151	17.0	
Diabetes	No	794	94.5	81,375	91.3	0.001
	Yes	47	5.6	7767	8.7	
COPD	No	819	97.5	84,226	94.5	0.001
	Yes	22	2.6	4916	5.5	
Hypertension	No	635	75.6	63,960	71.8	0.020
	Yes	206	24.5	25,182	28.2	

### Risk of death due to prostate cancer

**Fig. 1:**
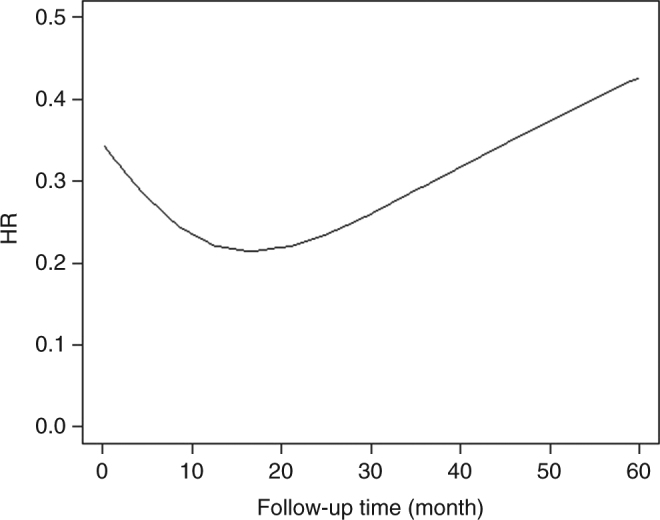
A nonparametric estimate of the hazard ratio as a function of follow-up time

The mortality rate in patients with prostate cancer was associated with a range of clinical and demographic factors (Supplementary Table 1). In Table 2, we show the adjusted HRs of prostate cancer mortality among patients with post-diagnostic use of cholera vaccine, as compared to patients who did not use cholera vaccine, which included all the potential confounding factors listed in Supplementary Table 1. After a mean age of 7.2 years and accumulated 6033 person-years of follow-up, 29 of them had died due to prostate cancer, giving a mortality rate of 4.8 per 1000 person-years, whereas the rate was 22.5 for those patients who did not use cholera vaccine. Use of cholera vaccine was associated with a decreased mortality rate, as compared to patients without cholera vaccination, with a crude HR of 0.33 (95% CI 0.23–0.47) and an adjusted HR of 0.57 (95% CI 0.40–0.82) after adjusting for a range of demographic and clinical factors. The results were similar when using patients who were not vaccinated and matched by a propensity score as the reference (Supplementary Table 3). The observed association was stratified by patient age at diagnosis, clinical stage, and disposable income. Reduced mortality associated with cholera vaccine was largely consistent, irrespective of these stratification factors. Although the point estimate was nearly identical among those patients diagnosed at a young age (<65-years old) and at an early stage (stages 1 and 2), the statistical significance was attenuated due to the limited number of outcomes. We also examined the risk of death due to prostate cancer among patients who received cholera vaccination before the diagnosis of cancer. The HR was 0.76 (95% CI 0.60–0.96) for those who received a vaccination as compared to the non-exposed cases. We further explored the actual HR as a function of the follow-up time (Figure 1). The figure indicated that the decreased HR was stronger after the vaccination of cholera vaccine, and it gradually became weaker after approximately 15 months.

**Table 2:** Hazard ratios of prostate cancer mortality among patients with post-diagnostic use of cholera vaccine as compared to patients without cholera vaccination

					Crude				Adjusted			
Characteristic	No. of patients	No. of person-years	No. of deaths	IR	HR	95% CI		*P* value	HR	95% CI		*P* value
Post-diagnostic use of cholera vaccine												
No	89,142	383,035	8622	22.5	Reference				Reference			
Yes	841	6033	29	4.8	0.33	0.23	0.47	<0.0001	0.57	0.40	0.82	0.00
Age at diagnosis												
<65	445	3344	9	2.7	0.45	0.23	0.86	0.02	0.57	0.30	1.11	0.09
≥65	396	2689	20	7.4	0.36	0.23	0.56	<0.0001	0.60	0.38	0.93	0.02
Stage at diagnosis												
Stages 1 and 2	738	5365	9	1.7	0.25	0.13	0.47	<0.0001	0.54	0.28	1.04	0.06
Stages 3 and 4	103	668	20	30.0	0.51	0.33	0.79	0.00	0.62	0.40	0.97	0.03
Income												
Lowest	71	499	1	2.0	0.09	0.01	0.62	0.01	0.20	0.03	1.40	0.10
Middle-low	129	935	2	2.1	0.12	0.03	0.46	0.01	0.20	0.05	0.82	0.02
Middle-high	213	1544	7	4.5	0.40	0.19	0.85	0.02	0.54	0.26	1.13	0.10
Highest	428	3055	19	6.2	0.80	0.51	1.26	0.32	0.73	0.46	1.16	0.18

*HR* indicates hazard ratio, and it was adjusted by age at diagnosis, year of diagnosis, birth country, highest education level, stage at diagnosis, income, region, chronic ischemic heart disease, chronic obstructive pulmonary disease, diabetes, and hypertension

*IR* indicates the incidence rate of prostate cancer mortality per 1000 person-years

### Risk of death due to all the causes

In Table 3, we show the risk of overall mortality among patients with prostate cancer who used cholera vaccine. The overall mortality rate was 9.4 per 1000 person-years for patients using cholera vaccine, whereas the rate was 48.3 for those who do not use cholera vaccine. A decreased overall mortality rate was found among patients using cholera vaccine, as compared with patients without cholera vaccination use, with a crude HR of 0.27 (95% CI 0.21–0.35) and an adjusted HR of 0.53 (95% CI 0.41–0.69). The decreased mortality rate was largely consistent, irrespective of age at diagnosis, clinical stage at diagnosis, and disposable income.

**Table 3:** Hazard ratios of overall mortality among patients with post-diagnostic use of cholera vaccine as compared to patients without cholera vaccination

Characteristic	No. of patients	No. of person-years	No. of deaths	IR	Crude				Adjusted			
					HR	95% CI		*P* value	HR	95% CI		*P* value
Post-diagnostic use of cholera vaccine												
No	89,142	383,035	18,496	48.3	Reference				Reference			
Yes	841	6033	57	9.4	0.27	0.21	0.35	<0.0001	0.53	0.41	0.69	<0.0001
Age at diagnosis												
<65	445	3344	18	5.4	0.46	0.29	0.74	0.001	0.61	0.38	0.97	0.04
≥65	396	2689	39	14.5	0.29	0.21	0.40	<0.0001	0.53	0.39	0.73	0.001
Stage at diagnosis												
Stages 1 and 2	738	5365	34	6.3	0.27	0.19	0.37	<0.0001	0.58	0.41	0.81	0.001
Stages 3 and 4	103	668	23	34.5	0.36	0.24	0.53	<0.0001	0.52	0.35	0.79	0.002
Income												
Lowest	71	499	4	8.0	0.14	0.05	0.38	<0.0001	0.37	0.14	0.99	0.04
Middle-low	129	935	9	9.6	0.21	0.11	0.41	<0.0001	0.38	0.20	0.73	0.003
Middle-high	213	1544	16	10.4	0.42	0.25	0.68	0.001	0.59	0.36	0.97	0.03
Highest	428	3055	28	9.2	0.53	0.36	0.77	0.001	0.58	0.40	0.84	0.003

*HR* indicates hazard ratio, and it was adjusted by age at diagnosis, year of diagnosis, birth country, highest education level, stage at diagnosis, income, region, chronic ischemic heart disease, chronic obstructive pulmonary disease, diabetes, and hypertension

*IR* indicates the incidence rate of prostate cancer mortality per 1000 person-years

### Sensitivity analyses

We performed sensitivity analyses by redefining exposure as one year after the administration of cholera vaccine and these gave similar results (Supplementary Table 1). Accounting for competing risks and removing cases identified by screening did not change the observed association. Patients who received antimalarial medications showed an increased cause-specific and overall mortality rate, as compared to patients who did not use antimalarial medications. Only 90 of them received both cholera vaccination and antimalarial medications (Supplementary Table 4).

## Discussion

This retrospective cohort study is, to the best of our knowledge, the first nationwide population-based study to explore whether use of cholera vaccine is associated with a reduced mortality rate in patients with prostate cancer. We found that patients with prostate cancer, who had used cholera vaccine after their cancer diagnosis, had a 43% decrease in prostate cancer mortality and a 47% decrease in overall mortality, as compared to patients who did not use cholera vaccine. The decreased mortality rate associated with cholera vaccination was largely consistent, irrespective of the patient’s age or tumor stage at diagnosis, and other potential confounders, including disposable income. Mortality reductions were also noted in several sensitivity analyses. Our findings suggest that cholera vaccine is associated with a reduced mortality rate in patients with prostate cancer^7–14^.

Studies examining the potential benefits of non-anticancer drugs or agents to patients with cancer, also called drug repurposing, are an attractive strategy for both academics and clinicians^15^. Such drugs, if successful, can offer additional treatment options to patients with cancer. A number of observational studies have been carried out to investigate the association between the use of non-anticancer drugs, such as statins and metformin, and different prostate cancer outcomes^16–22^. However, several potential biases could affect the observed associations and lead to inconsistent results. Common biases in these observation studies include immortal time bias, indication bias, health user bias, as well as no consideration of latency time windows between the use of non-anticancer drugs and outcomes^23^. In this population-based study, we have examined whether the use of cholera vaccine might be associated with a reduced mortality rate in patients with prostate cancer by taking into account all of the biases listed above. By using time-dependent Cox regression analyses, we could control the confounding by immortal time bias (main analysis). In addition, the influence of latency time on the observed associations was explored by defining the exposure to cholera vaccine as 1 year after vaccination (Sensitivity analysis 1). To control health user bias, we removed all prostate cancer cases that had been detected by screening (Sensitivity analysis 3), as men who undertook PSA screening might be associated with a high education and many types of healthy behavior^24^. In addition, we used patients without vaccination and matched them by a propensity score (no difference for all the clinical and demographic factors listed in Table 1, as compared to the study cohorts) as the reference; the data were largely similar. Furthermore, confounding by indication could not be totally ruled out, as we have no indications as to why some patients with prostate cancer received a vaccination for cholera. To control for this indication bias, we examined the mortality rate in patients with prostate cancer who used antimalarial medications; our hypothesis being that most Swedes who received either antimalarial medications or cholera vaccine might have traveled abroad (Sensitivity analysis 4). Although our study cannot eliminate all the confounding by unmeasured or misclassified prognostic factors that are associated with prostate cancer mortality, our study did give concrete evidence that use of cholera vaccine was associated with a risk reduction of cause-specific and overall mortality, as both the main analyses and several sensitivity analyses gave consistent results.

We acknowledge that the underlying mechanisms are largely unknown and further studies are required to explore the origins of these mechanisms. Previous animal studies have reported that cholera vaccine might have an antitumor function^7–14^. CTB can bind to the ganglioside on mammalian cells and is used as a component of oral cholera vaccine in Sweden^5^. CTB can induce an anti-inflammatory effect and regulate T-cell responses^5^. Animal studies showed that the administration of recombinant CTB can increase some immune cell populations, such as NK cells and macrophages^3,25^. In addition, some animal studies found that Th1 cells and CD8+ T cells can be promoted to prevent tumor growth after CTB vaccination inflates them^26^. In addition, available evidence suggests that the intestinal microbiota can affect how an individual responds to cholera vaccine^27^. Nicaraguan children who received an oral cholera vaccine have shown reduced antibody responses as compared to Swedish children^27^. A similar finding was noted among individuals who received a vaccination of *Shigella flexneri* with different immune responses among Bangladeshi adults and children, as compared to North American individuals^28^. These data suggest that the composition of intestinal microbiota may be a determining factor of vaccine efficacy, although other factors, such as socioeconomic conditions, nutritional status, and host genetics might also play a role. It remains unknown whether intestinal microbiota might be altered after oral cholera vaccination. Excessive bacterial growth was observed in the small intestine among children who received cholera vaccine^29^, which suggests that intestinal microbiota might be altered after oral cholera vaccination. As intestinal microbiota might play an important role regarding antitumor immunity^30,31^, cholera vaccination might protect against the progression of prostate cancer by alternating intestinal microbiota. Such knowledge is still lacking but highly needed in further studies. A study did find that cholera vaccination was associated with a lower mortality rate in patients with prostate cancer. Further studies, including well-designed cohort studies and randomized clinical trials, are warranted to confirm our research findings and to draw a causal relationship. In addition, whether the observed low mortality in patients who received cholera vaccination was due to improved immune function or altered intestinal microbiota should be explored further.

This study has a number of strengths and limitations, which should be kept in mind when interpreting the results. An important strength is that this population-based study has taken advantage of several nationwide registers in Sweden, which cover the whole Swedish population at a national level. The prospective study design and the completeness of the follow-up of patients are other major advantages of the present study. By using nationwide registers, our study could eliminate the recall bias and could minimize selection bias and misclassification. Some demographic factors and several clinical factors, such as the age of the patient at the time of diagnosis and tumor stage, which are the most significant factors that affect cancer mortality, could be identified from these registers and are included in the regression models. One limitation of this study is that the information on some individual-level risk factors, such as the PSA value, Gleason score, medical treatments, smoking status, and dietary factors are not available in our database, which may have partly confounded our conclusion. However, we could adjust the clinical stage at diagnosis in our regression models, which was the strongest prognostic factor for prostate cancer. In addition, Sweden is well-known for its universal healthcare for all Swedish citizens that is provided at a minimal cost. Discrepancy in medical treatment of prostate cancer is uncommon, as treatment decisions are made mainly based on clinical conditions instead of socioeconomic status. However, a recent study found that socioeconomic disparities in the management of men with a high risk of prostate cancer also exist in Sweden, but the discrepancy was minimal^32^. To account for the confounding by socioeconomic disparities in the management of prostate cancer, we have adjusted for place at living, disposable income, and education level in our regression model, which may partly minimize the confounding by these unmeasured factors^33,34^. In addition, we also stratified the analyses by disposable income, and the results were largely consistent. By using patients without vaccination and matching them by a propensity score as the reference, the observed findings were still significant, thus suggesting that the observed findings due to a discrepancy in socioeconomic status might be minimal. However, we acknowledge that the observed association might be a chance finding due to residual confounding. As the Swedish Prescribed Drug Register was established in July 2005, the study cohort was restricted to patients diagnosed with prostate cancer between July 2005 and December 2014. However, only 841 patients were found to have received cholera vaccination even in this nationwide population-based study. It is thus necessary to have an international collaboration to explore this subject further.

In summary, this population-based study shows that the use of cholera vaccine is associated with a decreased mortality in patients with prostate cancer. Risk reduction was largely consistent, irrespective of the age of the patients at the time of diagnosis and clinical stage. The findings from this study need to be confirmed by further randomized controlled studies to exclude the possibility of chance findings.

## Methods

### Study population

The Ethics Committee at Lund University approved this retrospective cohort study. This study was performed by combining several nationwide registers^35,36^. All patients with prostate cancer, who were diagnosed between July 2005 and December 2014, were identified from the Swedish Cancer Registry by using the 10th International Classification of Disease (ICD-10) code C61. The TNM staging system, including the size of tumor (T), nodal status (N), and the presence of metastatic disease (M), has been incorporated into the Swedish Cancer Registry since 2002. The stage at diagnosis of prostate cancer was determined by combining T, N, and M categories, which ranged from stage I (the least advanced) to stage IV (the most advanced). The stage was defined as follows: stage I (T1 or T2a N0 M0), stage II (T2b or T2c N0 M0), stage III (T3 N0 M0), and stage IV (T4 N1 M1)^37^.

Patients with prostate cancer were further linked to the Swedish Prescribed Drug Register to retrieve information about subsequent cholera vaccine use. The Swedish Prescribed Drug Register was established in July 2005 by the National Board of Health and Welfare and has almost 100% complete information about age and sex, as well as information regarding drug utilization and expenditures for all prescribed drugs in the entire Swedish population. The Anatomical Therapeutic Chemical (ATC) classification system was adopted in the register and used to classify all the drugs. Individuals who had been prescribed and dispensed with cholera vaccine were identified by using the ATC code J07AE01. In Sweden, cholera vaccine is composed of inactivated *Vibrio cholera* O1 and the recombinant CTB subunit, which is sold under the product name *Dukoral*. Our study included a total of 841 patients who used cholera vaccine and 89,142 patients who did not use cholera vaccine. To mitigate the possibility that patients using cholera vaccine might be healthier and be associated with a high socioeconomic status, multiple logistic regression analyses were conducted for the whole cohort and adjusted all the covariates listed in Table 1. We controlled the remaining imbalance by matching using the nearest-neighbor propensity scores, which is the probability of receiving cholera vaccination conditional on the observed baseline characteristics. We did a 1-to-3 match so that the two groups of patients had the same/similar propensity score (Supplementary Table 2). After being matched by propensity scores, the propensity-matched pairs (patients receiving cholera vaccination vs. patients without vaccination) did not have any significant difference in the demographic and clinical characteristics listed in Table 1.

Patients with prostate cancer were further combined with other registers, such as with the Total Population Register and with the Population Housing Census to obtain information on individual-level characteristics. Patients who died during the study period were identified from the Cause of Death Register. All linkages were performed using individual national identification numbers, which were replaced with serial numbers in order to preserve anonymity. Informed consent was not necessary in this study, as we used register-based data and replaced individuals’ identification numbers to ensure anonymity.

### Study outcome

From the Cause of Death Register, we identified all patients with prostate cancer who died between July 2005 and December 2015. The primary outcome was death due to prostate cancer as the primary cause of death (ICD code: C61). The secondary outcome was death due to all the causes (ICD codes: A00-Z99).

### Statistical analysis

To account for immortal time bias, time-dependent Cox regression was used to calculate HRs and 95% CIs for prostate cancer mortality and all-cause mortality. Use of cholera vaccine after the diagnosis of prostate cancer was treated as a time-dependent variable in the model, thus allowing patients who moved from a follow-up period of non-exposure (from diagnosis of prostate cancer to the administration of cholera vaccine) to a period of exposure (being vaccinated with cholera vaccine and thereafter for the remainder of follow-up). Several clinical and demographic factors listed in Supplementary Table 1, which were associated with mortality in patients with prostate cancer, were included in the regression model to account for their potential confounding effects. The model included patient age at diagnosis of prostate cancer, year at diagnosis of prostate cancer, individual disposable income (lowest, middle-low, middle-high, and highest), region at diagnosis (big cities, southern and northern Sweden), stage of prostate cancer (stages 1, 2, 3, and 4), country of birth (Sweden, European countries, and others), the highest educational level (1–9, 10–11, and 12+), and comorbidities (yes or no).

We censored individuals (i.e., treated them as no longer under observation or at a risk of the study outcome) at the time of death from any cause, at the end of the follow-up period (December 31, 2015), or at the time of emigration, whichever came first. The proportional hazards assumption was tested using cumulative martingale residuals, as shown in Supplementary Table 5. If the variables did not meet the assumption, we used a stratification model and they were treated as stratification variables. All analyses were performed using SAS version 9.2 (SAS Institute, Cary, NC, USA).

### Sensitivity analyses

We performed several sensitivity analyses to reduce the possibility of chance findings. First, exposure to cholera vaccine was lagged one year after the administration of cholera vaccine; given that short duration of exposures are unlikely to be associated with the mortality outcomes^38^. Second, the effect of competing risks as a result of death from other causes was evaluated by using the subdistribution hazards model proposed by Fine and Gray^39^. Third, we removed all prostate cancer cases diagnosed by PSA screening (defining screening-detected prostate cancer by using the clinical stage of T1c) to exclude its potential effect on the observed associations, because patients with screening-detected prostate cancer might be associated with health behaviors leading to healthy user bias. Fourth, we controlled for confounding by indication (indication bias) by examining the mortality in patients with prostate cancer who had used antimalarial medications, given the probability that most Swedes who received antimalarial medications or cholera vaccine might have traveled abroad. Fifth, we calculated the HRs using patients without vaccination and matched them by a propensity score as the reference (Supplementary Table 1).

### Data availability

All relevant data are available from the authors upon reasonable request.

## Electronic supplementary material

Supplementary Information

Peer Review File
