# Supplementary material for: Association between post-diagnostic use of cholera vaccine and risk of death in prostate cancer patients
Source: Nat Commun. 2018 Jun 18;9:2367. doi: 10.1038/s41467-018-04814-4 (PMC6006429; doi:10.1038/s41467-018-04814-4)
Supplement: Supplementary file 1 — Supplementary Information [file 41467_2018_4814_MOESM1_ESM.pdf]

Association between post-diagnostic use of cholera vaccine and risk of death in prostate cancer patients

Ji et al

Supplementary Table 1. Associations of prostate cancer mortality with clinical and demographic characteristics

Characteristic	No. patients	No. of person-years	No. of death	IR	HR	95%CI		P value
Age at diagnosis								
<60	12253	59981	514	8.6		Reference		
60-64	16192	80092	762	9.5	1.11	0.99	1.24	0.067
65-69	21255	93194	1183	12.7	1.48	1.33	1.64	<.0001
70+	40283	155799	6192	39.7	4.62	4.22	5.05	<.0001
Year at diagnosis								
2005-2009	37512	248699	5741	23.1		Reference		
2010-2014	52471	140368	2910	20.7	0.81	0.77	0.85	<0.0001
Highest education, years								
1-9	30195	130632	4162	31.9		Reference		

10-11	33915	146503	2805	19.1	0.60	0.57	0.63	<.0001
12+	25873	111931	1684	15.0	0.46	0.43	0.48	<.0001
Birth country								
Sweden	81974	354884	7921	22.3		Reference		
European countries	6381	27564	629	22.8	1.02	0.94	1.11	0.6033
Others	1628	6618	101	15.3	0.68	0.56	0.83	0.0001
Income								
Lowest	22941	94587	3245	34.3		Reference		
Middle-low	22357	96240	2660	27.6	0.81	0.77	0.85	<.0001
Middle-high	22342	97804	1639	16.8	0.49	0.46	0.52	<.0001
Highest	22343	100435	1107	11.0	0.32	0.30	0.35	<.0001
Region								
Big cities	42634	187666	3532	18.8		Reference		
Southern Sweden	29486	128057	3030	23.7	1.26	1.20	1.32	<.0001

Northern Sweden	17863	73343	2089	28.5	1.51	1.43	1.59	<.0001
Stage at diagnosis								
Stage 1	43782	203721	969	4.8		Reference		
Stage 2	25726	114529	1584	13.8	2.92	2.70	3.17	<.0001
Stage 3	11827	47288	2071	43.8	9.34	8.66	10.08	<.0001
Stage 4	8648	23528	4027	171.2	37.70	35.13	40.46	<.0001
Chronic ischemic heart disease								
No	74741	322143	6617	20.5		Reference		
Yes	15242	66924	2034	30.4	1.48	1.41	1.56	<.0001
Diabetes								
No	82169	354080	7493	21.2		Reference		
Yes	7814	34987	1158	33.1	1.57	1.47	1.67	<.0001
Chronic obstructive pulmonary disease								

No	85045	366882	7987	21.8		Reference		
Yes	4938	22185	664	29.9	1.38	1.27	1.49	<.0001
Hypertension								
No	64595	269347	5469	20.3		Reference		
Yes	25388	119720	3182	26.6	1.32	1.26	1.37	<.0001

Supplementary Table 2. Baseline demographic and clinical characteristics of patients with prostate cancer being vaccinated with cholera vaccine and patients without vaccination and matched by propensity score

	Users of cholera vaccine		Patients without vaccination and matched using propensity score		
Characteristic	No. of patients	%	No. of patients	%	p value
Overall	831	100	2427	100	
Age at diagnosis					
<60	221	26.6	666	27.4	0.55
60-64	218	26.2	652	26.9	
65-69	252	30.3	674	27.8	
70+	140	16.8	435	17.9	
Median age, years	64		64		
Year at diagnosis					
2005-2009	619	74.5	1817	74.9	0.83
2010-2014	212	25.5	610	25.1	

Median	2008		2008		
Highest education, years					
1-9	152	18.3	452	18.6	0.56
10-11	298	35.9	919	37.9	
12+	381	45.8	1056	43.5	
Birth country					
Sweden	787	94.7	2309	95.1	0.79
European countries	36	4.3	100	4.1	
Others	8	1.0	18	0.7	
Income					
Lowest	67	8.1	204	8.4	0.12
Middle-low	125	15.0	398	16.4	
Middle-high	211	25.4	691	28.5	
Highest	428	51.5	1134	46.7	

Region					
Big cities	447	53.8	1346	55.5	0.34
Southern Sweden	249	30.0	737	30.4	
Northern Sweden	135	16.2	344	14.2	
Stage at diagnosis					
Stage 1	529	63.7	1599	65.9	0.50
Stage 2	200	24.1	573	23.6	
Stage 3	67	8.1	171	7.0	
Stage 4	35	4.2	84	3.5	
Chronic ischemic heart disease					
No	740	89.0	2189	90.2	0.34
Yes	91	11.0	238	9.8	
Diabetes					

No	787	94.7	2321	95.6	0.27
Yes	44	5.3	106	4.4	
Chronic obstructive pulmonary disease					
No	809	97.4	2389	98.4	0.05
Yes	22	2.6	38	1.6	
Hypertension					
No	628	75.6	1847	76.1	0.75
Yes	203	24.4	580	23.9	

Supplementary Table 3. Sensitivity analyses of the adjusted HR between postdiagnostic use of cholera vaccine and the risk of death

	Cause specific mortality				Overall mortality			
Characteristic	HR	95%CI		P value	HR	95%CI		P value
Sensitivity analysis 1	0.46	0.31	0.68	<0.0001	0.54	0.43	0.69	<0.0001
Sensitivity analysis 2	0.50	0.33	0.76	0.001		NA		
Sensitivity analysis 3	0.49	0.31	0.74	0.001	0.53	0.39	0.71	<0.0001
Sensitivity analysis 4	1.52	1.35	1.71	<0.0001	1.46	1.36	1.57	<0.0001
Sensitivity analysis 5	0.54	0.35	0.83	<0.0001	0.53	0.39	0.70	<0.0001

Sensitivity analysis 1: One year of latency time windows between cholera vaccination and mortality
Sensitivity analysis 2: Fine & Gray competing risk model
Sensitivity analysis 3: Removing patients with prostate cancer who were identified by screening
Sensitivity analysis 4: Patients with prostate cancer who received antimalarial treatment as compared to non-exposed cases
Sensitivity analysis 5: Using patients without vaccination and matched by propensity score as the reference.

Supplementary Table 4. Cross tabulation of exposure to cholera vaccine and antimalarial medications

		Antimalarial medication			
		Yes		No	
		No.	%	No.	%
Cholera vaccine	Yes	90	0.10	751	0.83
	No	3549	3.94	85593	95.12

Supplementary Table 5. Test for proportional hazards assumption using cumulative martingale residuals

Variables	P value
Education	0.217
Birth countries	0.285
Clinical stage	<.0001
Income	0.002
Region	0.006
Chronic ischemic heart disease	0.37
Chronic obstructive pulmonary disease	0.141
Diabetes	0.399
Hypertension	0.746
