# Supplementary material for: Association between post-diagnostic use of cholera vaccine and risk of death in prostate cancer patients
Source: Nat Commun. 2018 Jun 18;9:2367. doi: 10.1038/s41467-018-04814-4 (PMC6006429; doi:10.1038/s41467-018-04814-4)
Supplement: Supplementary file 2 — Peer Review File [file 41467_2018_4814_MOESM2_ESM.pdf]

Reviewers' comments:

Reviewer #1 (Remarks to the Author):

The manuscript reports an association between cholera vaccine administration and reduction in prostate cancer mortality. The study cohort featured prostate cancer cases drawn from the Swedish Cancer Registry (2005-2014; n = 827 who had used vaccine and 3944 who had not), with linkage to the Swedish Prescribed Drug Register for determination of cholera vaccine use. The cholera vaccine preferentially administered featured inactivated Vibrio cholera O1 and recombinant cholera toxin B subunit given orally. The case-case study design employed matching 5:1 for age at diagnosis and tumor stage. Attempts to account for several confounding influences were also undertaken.

Among the men with prostate cancer who were vaccinated, 29 (3.5%) died as a result of prostate cancer. When compared to men with prostate cancer left unvaccinated, use of cholera vaccine was associated with a reduced risk of prostate cancer death, with a hazard ratio of 0.5. With the low number of deaths, the phenomenon was more apparent among older men with higher stage disease. Diminished overall mortality was also evident, with a hazard ratio of 0.6.

With these data, the authors concluded/conjectured that components of the cholera vaccine might have an anti-tumor effect. The association reported is unquestionably provocative. The idea that this might reflect a direct anti-tumor action of vaccine components is not at all convincing- and may detract from the main manuscript findings. One downside of searching for disease associations in an exploratory mode is that the provisional conclusions reached can be undermined by bias. And, although the authors pursued several analyses to address this concern, bias remains a significant worry.

What kinds of unrecognized biases could be associated with cholera vaccine use? The World Health Organization (WHO) recommends vaccination (in addition to food hygiene) for travel to areas of the world where cholera is endemic. What are the attributes of men who travel to regions of the world with endemic cholera versus non-travelers? The oral vaccine is typically administered in 2-3 doses and can provide some level of protection, both against cholera and against entertoxin-producing E. coli, for months-years. From the data presented no more than 17% of men with prostate cancer received vaccine (and probably substantially less, though this statistic was not included in the paper). One analysis not presented was the use of other 'traveler' vaccines, such as for hepatitis A, or the use of antibiotic prophylaxis.

The authors should consider the possibility that the intestinal microbiome could be what is altered by oral cholera vaccines. The gut microbiome has been found to mediate many different systemic processes, including anti-tumor immunity, fatty liver, etc. Are any consequences of cholera vaccine administration on microbiome components/bacteria quora known?

The Dukoral vaccine used in Sweden contains 1 mg recombinant cholera toxin B subunit (similar to the native protein with an additional 7 N-terminal amino acids) and 25 X 10e9 each of four killed cholera bacterial strains. The toxin B subunit is the component that binds to the GM1-ganglioside in intestinal cells; the A subunit contains the toxin activity. The cholera toxin B subunit has demonstrated activity as a vaccine adjuvant generally, though not so much so when administered orally. Oral administration of the B subunit in human clinical trials has modified the natural history of inflammatory bowel disease, emboldening the notion that the toxin subunit might aid in promotion of oral tolerance to treat autoimmunity. What is not clear is whether the cholera toxin B subunit has much in the way of oral bioavailability. Differences in immune responses from intranasal, oral, and injected cholera toxin B subunit hint that the protein may not appear at significant levels in the

circulation. As such, killing of prostate cancer cells by orally administered cholera toxin B subunit seems very unlikely.

The near equivalent effect of cholera vaccine administration on overall mortality also argues against a direct effect of vaccine components on prostate cancer cells. Most men with prostate cancer die of something other than prostate cancer. A cholera vaccine effect on cardiovascular mortality is much more easily explained by anti-inflammatory consequences of vaccine administration.

Reviewer #2 (Remarks to the Author):

The report by Dr. Ji and colleagues uses national databases in Sweden to test the hypothesis that post-diagnostic use of cholera vaccine is associated with risk of death among patients with prostate cancer. Results suggest risk of death from prostate cancer and overall mortality is decreased among prostate cancer patients who had been prescribed the anti-cholera vaccine.

There are a number of strengths evident in this report. The country has terrific resources that enable the facile testing of the hypothesis, including the Swedish Cancer Registry and the Swedish Prescribed Drug Register. The health system should enable uniform access to care. The findings are provocative.

The manuscript presents this as the first prospective study to show an association on the cholera vaccine and prostate cancer mortality. I couldn't find ANY studies on this topic, other than a report by the same group on the same exposure but for colon cancer. Given the novelty of this finding, readers will be particularly skeptical, requiring a rigorous review of the methods and a balanced interpretation of the results. Based on the materials presented, I'm not completely convinced that the findings are valid, based in part on the study design, the analysis, the lack of treatment data, and the potential for uncontrolled confounding. The justification for the analysis/biological plausibility is superficial and the biggest weakness. One suggestion is to strengthen the introduction with a paragraph on how the cholera B subunit is being used for therapeutic vaccines. That line of research is much more specific than what is currently cited.

Design and analysis issues:

• The investigators have access to the entire country's data, correctly describe this as a cohort, yet analyze as a nested case-control study. The justification for that is unclear to this reviewer. Matching can introduce bias, and although it may be efficient in some regards, since the data are available electronically, the only additional effort/cost would be computation time. However, treating as a proper cohort analysis will strengthen the design and increase confidence that the results are valid.

• The timing of the vaccine relative to diagnosis and relative to the endpoints needs more clear presentation. The time-dependency appears to be captured, but what cannot be discerned is the actual hazard ratio over time. A figure would be very instructive: is the separation of mortality immediately apparent? Does the apparent effect change over time? Is there a lag period? A more rigorous analysis of this issue is essential.

• Why were the years of observation restricted to July 2005 and December 2014? This should be included in the methods, and any potential weaknesses in the approach could be addressed in the discussion.

• What is known about vaccination prior to diagnosis? Are those records not available? Presumably men got vaccinated at different time periods post diagnosis. The distribution of the lag time between diagnosis and vaccination, and vaccination to death, must be presented to the reviewers. A simple

dichotomy of exposed/not exposed is insufficient.

• How was stage handled in the analysis? Grouped as in Table 2 or with the stages independent?

• Similar concerns about how income was categorized. Categorical or continuous? The latter is preferred.

• In the United States, there may be multiple causes of death listed on the death certificate. Is the distinction here prostate cancer as the primary cause versus not noted anywhere? If a secondary cause, how are they counted?

• A list of variables are described as confounders. They should be described as potential confounders. A supplementary table could be provided to allow the readers to discern for themselves the impact: are they associated with exposure (vaccine) and mortality?

Lack of treatment data:
• I do not subscribe to the statement that stage data are sufficient.

• There is a significant difference between the men who got vaccines and those who did not with regard to income. Presumably that relates to treatment, but these data show that income certainly relates to overall mortality rates.

• The authors acknowledge this as a limitation, and no observational study is perfect, but it places greater importance on other aspects of the study design and the biological plausibility.

Uncontrolled confounding/biologic plausibility:

• The income differences between men who got the vaccine and those who did not is striking. Perhaps that contributes to differences in care. Based on the literature, income is related to decreased overall mortality.

• The exposed cases are also generally more healthy than the non-exposed cases. This could be an indication of their ability to endure more aggressive treatment contributing to the lower prostate cancer mortality, but also the overall lower mortality.

• This appears to play out when comparing prostate cancer mortality and overall mortality. The biologic plausibility presented by the authors is very cancer-specific. Why then would the effect be evident for total mortality too? It strikes me as reflecting the possibility that the exposed cases were just generally healthier, richer, and they had the opportunity to travel to where an anti-cholera vaccine would be warranted. Thus, it is a true, true but unrelated scenario.

• The analysis of anti-malarial drugs was presented as a sensitivity analysis. What is the concordance with anti-cholera vaccine? It is difficult to interpret these findings.

Reviewers' comments:

Reviewer #1 (Remarks to the Author):

#1. The manuscript reports an association between cholera vaccine administration and reduction in prostate cancer mortality. The study cohort featured prostate cancer cases drawn from the Swedish Cancer Registry (2005-2014; n = 827 who had used vaccine and 3944 who had not), with linkage to the Swedish Prescribed Drug Register for determination of cholera vaccine use. The cholera vaccine preferentially administered featured inactivated Vibrio cholera O1 and recombinant cholera toxin B subunit given orally. The case-case study design employed matching 5:1 for age at diagnosis and tumor stage. Attempts to account for several confounding influences were also undertaken.

Among the men with prostate cancer who were vaccinated, 29 (3.5%) died as a result of prostate cancer. When compared to men with prostate cancer left unvaccinated, use of cholera vaccine was associated with a reduced risk of prostate cancer death, with a hazard ratio of 0.5. With the low number of deaths, the phenomenon was more apparent among older men with higher stage disease. Diminished overall mortality was also evident, with a hazard ratio of 0.6.

#1. Response: We appreciate the positive responses from the reviewer. We have now recalculated the HR using all patients with prostate cancer but without cholera vaccine as the reference as suggested by the reviewer 2 (See #9). The results were very similar. In addition, we used propensity score matched controls as the reference to control for the difference of the clinical and demographic factors between the study cohorts and the controls, and the results agreed with the main findings.

#2. With these data, the authors concluded/conjectured that components of the cholera vaccine might have an anti-tumor effect. The association reported is unquestionably provocative. The idea that this might reflect a direct anti-tumor action of vaccine components is not at all convincing- and may detract from the main manuscript findings. One downside of searching for disease associations in an exploratory mode is that the provisional conclusions reached can be undermined by bias. And, although the authors pursued several analyses to address this concern, bias remains a significant worry.

#2 Response: We agree with the reviewer that bias might be a concern in this observation study. We have tried a few extra analyses, and used all patients with prostate cancer but without cholera vaccine as the controls. We also used propensity score matched controls as the reference. In addition, we have tone down the conclusion, and discussed the observation in more detail in the limitation section of the discussion.

#3. What kinds of unrecognized biases could be associated with cholera vaccine use? The World Health Organization (WHO) recommends vaccination (in addition to food hygiene) for travel to areas of the world where cholera is endemic. What are the attributes of men who travel to regions of the world with endemic cholera versus non-travelers? The oral vaccine is typically administered in 2-3 doses and can provide some level of protection, both against cholera and against entertoxin-producing E. coli, for months-years. From the data presented no more than 17% of men with prostate cancer received vaccine (and probably substantially less, though this statistic was not included in the paper). One analysis not presented was the use of other 'traveler' vaccines, such as for hepatitis A, or the use of antibiotic prophylaxis.

#3 Response: An important comment we carefully analyzed and found that most patients who received hepatitis A vaccination did also receive cholera vaccine in Sweden. We found only 15 patients who received hepatitis A vaccine but not with cholera vaccine. Instead we looked

at the HR in patients who received anti-malaria treatment, as shown in sensitivity analyses. The data from the sensitivity analyses suggest that indication bias might be minimal.

#4. The authors should consider the possibility that the intestinal microbiome could be what is altered by oral cholera vaccines. The gut microbiome has been found to mediate many different systemic processes, including anti-tumor immunity, fatty liver, etc. Are any consequences of cholera vaccine administration on microbiome components/bacteria quora known?

#4 Response We agree with the reviewer that intestinal microbiome might play an important role for the observed association, and we have discussed it in the discussion section.

#5 The Dukoral vaccine used in Sweden contains 1 mg recombinant cholera toxin B subunit (similar to the native protein with an additional 7 N-terminal amino acids) and 25 X 10e9 each of four killed cholera bacterial strains. The toxin B subunit is the component that binds to the GM1-ganglioside in intestinal cells; the A subunit contains the toxin activity. The cholera toxin B subunit has demonstrated activity as a vaccine adjuvant generally, though not so much so when administered orally. Oral administration of the B subunit in human clinical trials has modified the natural history of inflammatory bowel disease, emboldening the notion that the toxin subunit might aid in promotion of oral tolerance to treat autoimmunity. What is not clear is whether the cholera toxin B subunit has much in the way of oral bioavailability. Differences in immune responses from intranasal, oral, and injected cholera toxin B subunit hint that the protein may not appear at significant levels in the circulation. As such, killing of prostate cancer cells by orally administered cholera toxin B subunit seems very unlikely.

#5 Thanks the reviewer for pointing out this. We have now discussed the observed association in detail, and tried to interpret the observed association by intestinal microbiome and altered immune function.

#6 The near equivalent effect of cholera vaccine administration on overall mortality also argues against a direct effect of vaccine components on prostate cancer cells. Most men with prostate cancer die of something other than prostate cancer. A cholera vaccine effect on cardiovascular mortality is much more easily explained by anti-inflammatory consequences of vaccine administration.

6 Response: We have calculated the risk of death due to myocardial infarction, and chronic ischemic heart diseases. However, none of them showed a significant result due to the small number of deaths. However, the non-significant lower mortality due to myocardial infarction, and chronic ischemic heart diseases might be associated with anti-inflammatory consequences of vaccine administration.

Reviewer #2 (Remarks to the Author):

#7 The report by Dr. Ji and colleagues uses national databases in Sweden to test the hypothesis that post-diagnostic use of cholera vaccine is associated with risk of death among patients with prostate cancer. Results suggest risk of death from prostate cancer and overall mortality is decreased among prostate cancer patients who had been prescribed the anti-cholera vaccine.

There are a number of strengths evident in this report. The country has terrific resources that enable the facile testing of the hypothesis, including the Swedish Cancer Registry and the

Swedish Prescribed Drug Register. The health system should enable uniform access to care. The findings are provocative.

#7 Response: We appreciate the positive response from the reviewer.

#8 The manuscript presents this as the first prospective study to show an association on the cholera vaccine and prostate cancer mortality. I couldn't find ANY studies on this topic, other than a report by the same group on the same exposure but for colon cancer. Given the novelty of this finding, readers will be particularly skeptical, requiring a rigorous review of the methods and a balanced interpretation of the results. Based on the materials presented, I'm not completely convinced that the findings are valid, based in part on the study design, the analysis, the lack of treatment data, and the potential for uncontrolled confounding. The justification for the analysis/biological plausibility is superficial and the biggest weakness. One suggestion is to strengthen the introduction with a paragraph on how the cholera B subunit is being used for therapeutic vaccines. That line of research is much more specific than what is currently cited.

#8 Response: We agree with the reviewer and we have rewritten the introduction section, and included more information about the biological plausibility of cholera vaccine on the observed outcomes based on current literature. In addition, we pointed out that cholera vaccine is currently used only as anti-cholera infection, although it might be repositioned as adjuvant therapy for patients with prostate cancer based on its ability to regulate immune function.

Design and analysis issues:

#9 • The investigators have access to the entire country's data, correctly describe this as a cohort, yet analyze as a nested case-control study. The justification for that is unclear to this reviewer. Matching can introduce bias, and although it may be efficient in some regards, since

the data are available electronically, the only additional effort/cost would be computation time. However, treating as a proper cohort analysis will strengthen the design and increase confidence that the results are valid.

#9 Response: We have now included all patients with prostate cancer but without cholera vaccine as the controls. The results were very similar.

#10 • The timing of the vaccine relative to diagnosis and relative to the endpoints needs more clear presentation. The time-dependency appears to be captured, but what cannot be discerned is the actual hazard ratio over time. A figure would be very instructive: is the separation of mortality immediately apparent? Does the apparent effect change over time? Is there a lag period? A more rigorous analysis of this issue is essential.

#10 Response: We thanks the reviewer to point out this. We have now presented a nonparametric estimate of the hazard ratio as a function of follow-up time as shown in the new Figure 1.

11 • Why were the years of observation restricted to July 2005 and December 2014? This should be included in the methods, and any potential weaknesses in the approach could be addressed in the discussion.

#11 Response: As the Swedish Prescription Register was started in July 2005, thus only patients with prostate cancer diagnosed after that were included in the current study. We have discussed this limitation of this study in the discussion section.

#12 • What is known about vaccination prior to diagnosis? Are those records not available? Presumably men got vaccinated at different time periods post diagnosis. The distribution of the lag time between diagnosis and vaccination, and vaccination to death, must be presented to the reviewers. A simple dichotomy of exposed/not exposed is insufficient.

#12 Response: We have now presented lag time between diagnosis and vaccination in Table 1. We also calculated the HR among those patients who received cholera vaccine before the

diagnosis, and a significant decreased mortality due to prostate cancer was noted.

#13 • How was stage handled in the analysis? Grouped as in Table 2 or with the stages independent?

Response#13: Stage at diagnosis was handled as independent variable.

#14 • Similar concerns about how income was categorized. Categorical or continuous? The latter is preferred.

#14 Response As income did not meet normal distribution, we thus included it as categorical variable, as shown in supplementary Table 1.

#15• In the United States, there may be multiple causes of death listed on the death certificate. Is the distinction here prostate cancer as the primary cause versus not noted anywhere? If a secondary cause, how are they counted?

#15 Response We only looked the risk of death due to prostate cancer as the primary cause in the current study. Thus we did not include the secondary causes of mortality only the primary cause.

#16• A list of variables are described as confounders. They should be described as potential confounders. A supplementary table could be provided to allow the readers to discern for themselves the impact: are they associated with exposure (vaccine) and mortality?

#16 Response Thanks the reviewer for pointing out this. We have now looked the association of these potential confounders with both exposure (Table 1) and mortality (supplementary Table 1).

Lack of treatment data:

•#17 I do not subscribe to the statement that stage data are sufficient. There is a significant difference between the men who got vaccines and those who did not with regard to income.

Presumably that relates to treatment, but these data show that income certainly relates to overall mortality rates. The authors acknowledge this as a limitation, and no observational study is perfect, but it places greater importance on other aspects of the study design and the biological plausibility.

•#17 Response We agree with the reviewer that stage data are sufficient. We have now discussed this in detail. Sweden is well known for its universal healthcare for all Swedish citizens at a minimal cost. Discrepancy in medical treatment of prostate cancer is probably very uncommon. In addition, we have stratified the analyses by disposable income, and the results were largely consistent.

Uncontrolled confounding/biologic plausibility:

#18• The income differences between men who got the vaccine and those who did not is striking. Perhaps that contributes to differences in care. Based on the literature, income is related to decreased overall mortality.

#18 Response Please see also our response above # 17. We have we stratified the analyses by disposable income, and the results were largely consistent. In addition, we have used propensity score matched controls (no difference of the clinical and demographic factors as compared to the study cohorts) as the reference, and the observed findings were largely similar.

#19 • The exposed cases are also generally more healthy than the non-exposed cases. This could be an indication of their ability to endure more aggressive treatment contributing to the lower prostate cancer mortality, but also the overall lower mortality.

#19 Response We agree with reviewer that exposed cases were generally healthier than controls. We have tried to control confounding by a few extra analyses, and adjusted all the potential confounding factors in the model, including comorbidities, and socioeconomic status.

#20• This appears to play out when comparing prostate cancer mortality and overall mortality. The biologic plausibility presented by the authors is very cancer-specific. Why then would the effect be evident for total mortality too? It strikes me as reflecting the possibility that the exposed cases were just generally healthier, richer, and they had the opportunity to travel to where an anti-cholera vaccine would be warranted. Thus, it is a true, true but unrelated scenario.

#20 Response: We have discussed the biologic plausibility in detail. The potential mechanism might be due to intestinal microbiota or altered immune function. In addition, we have used propensity score matched controls (no difference of the clinical and demographic factors as compared to the study cohorts) as the reference, and the observed findings were largely similar.

#21• The analysis of anti-malarial drugs was presented as a sensitivity analysis. What is the concordance with anti-cholera vaccine? It is difficult to interpret these findings.

#21 Response The analysis of anti-malarial drugs was used to deal with indication bias as we have no information why patients received cholera vaccine, but individuals received cholera vaccine and anti-malarial drugs might travel abroad.

Reviewers' comments:

Reviewer #1 (Remarks to the Author):

The key issue for this provocative study is whether a lower risk of prostate cancer mortality accompanying cholera vaccine use might reflect some sort of bias, rather than highlight a beneficial action of cholera vaccination per se on the natural history of prostate cancer.

The revised manuscript now contains more efforts to address sources of bias, and a broader discussion of potential mechanisms by which cholera vaccines might affect physiology and health, such as through perturbing the intestinal microbiome. There do not seem to be any additional ways in which the current dataset can be usefully interrogated further.

Nonetheless, skeptical readers may still be difficult to convince of any causal relationship between cholera vaccine administration and prostate cancer outcome. Clearly, some sort of intervention study may be required.

Reviewer #2 (Remarks to the Author):

The authors have addressed most of my previous concerns. There are a few lingering issues, and some introduced by the revision and additional analyses.

A consistent issue that needs to be corrected throughout is the nomenclature and use of the term 'controls'. Although the authors can certainly define a term and use as they wish, most readers will be familiar with the traditional usage, which is a person free of the disease. Here the authors use to represent cases who have not been vaccinated. That is confusing. In fact, there are other instances in the text for other analyses that perpetuate the concerns.

Although the idea to generate the propensity score is a seemingly good idea, the way it is presented is a bit confusing. First, line 103 defines a propensity score as the probability of treatment assignment. I believe they mean vaccine exposure classification? That the authors accepted my suggestion to use all cases during the study period (a proper cohort, with exposed versus non-exposed representing use of the cholera vaccine) is a nice revision, but the reference to "controls" in light of the cohort analysis makes little sense. For example, Line 70 refers to matched controls. Again on line 98 there is reference to controls. Some readers might interpret that to be cancer-free individuals, not prostate cancer cases unexposed to the vaccine. In fact, line 105 leads me to wonder who exactly is being compared. "Study cohorts versus matched controls". Controls who are healthy and neighbors (residence-wise) to cases? The table leads me to believe that the authors are using a subset of cases (not exposed) rather than neighborhood controls (without cancer), but more precise language is needed.

The analysis of pre-diagnosis exposure to the vaccine is a nice addition. Again, I encourage the use of "non-exposed cases" rather than "controls" for the sentence beginning on line 185.

The sentence on line 183 that begins with "However" focuses on the statistical significance, but in fact the point estimates of effect are nearly identical. That should be stated first, followed by the observation that statistical significance was attenuated, and the attribution to small sample size is appropriate.

Figure 1. Y axis should be HR (in time not needed).

Response #21 about anti-malarial drug concordance was not adequately addressed. It can be in at least two ways. A simple cross-tabulation of the exposures, or an analysis that considers the combination of the two exposures: no/no, yes/no, no/yes, yes/yes.

The authors need to more strongly acknowledge that the findings are chance, perhaps due to residual confoounding, beyond the minimal comment that starts on line 303. For starters, line 144 should state that the sensitivity analyses were performed to "reduce" not "exclude" the possibility of chance findings.

Line 137 says that the proportional hazards assumption was tested, but the results are never given. They should be added.

Reviewers' comments:

Reviewer #1 (Remarks to the Author):

The key issue for this provocative study is whether a lower risk of prostate cancer mortality accompanying cholera vaccine use might reflect some sort of bias, rather than highlight a beneficial action of cholera vaccination per se on the natural history of prostate cancer.

The revised manuscript now contains more efforts to address sources of bias, and a broader discussion of potential mechanisms by which cholera vaccines might affect physiology and health, such as through perturbing the intestinal microbiome. There do not seem to be any additional ways in which the current dataset can be usefully interrogated further.

Nonetheless, skeptical readers may still be difficult to convince of any causal relationship between cholera vaccine administration and prostate cancer outcome. Clearly, some sort of intervention study may be required.

Response: We appreciate the positive responses from the reviewer. We acknowledged that chance finding might be possible due to residual confounding as shown in page 14. We also pointed out that intervention studies are needed to confirm our observation before draw any causal conclusion as shown in page 13.

Reviewer #2 (Remarks to the Author):

The authors have addressed most of my previous concerns. There are a few lingering issues, and some introduced by the revision and additional analyses.

Response: We appreciate the positive responses from the reviewer.

A consistent issue that needs to be corrected throughout is the nomenclature and use of the term ‘controls’. Although the authors can certainly define a term and use as they wish, most

readers will be familiar with the traditional usage, which is a person free of the disease. Here the authors use to represent cases who have not been vaccinated. That is confusing. In fact, there are other instances in the text for other analyses that perpetuate the concerns.

Response: Thank the review for pointing out this. We have now revised the text accordingly.

Although the idea to generate the propensity score is a seemingly good idea, the way it is presented is a bit confusing. First, line 103 defines a propensity score as the probability of treatment assignment. I believe they mean vaccine exposure classification? That the authors accepted my suggestion to use all cases during the study period (a proper cohort, with exposed versus non-exposed representing use of the cholera vaccine) is a nice revision, but the reference to “controls” in light of the cohort analysis makes little sense. For example, Line 70 refers to matched controls. Again on line 98 there is reference to controls. Some readers might interpret that to be cancer-free individuals, not prostate cancer cases unexposed to the vaccine. In fact, line 105 leads me to wonder who exactly is being compared. “Study cohorts versus matched controls”. Controls who are healthy and neighbors (residence-wise) to cases? The table leads me to believe that the authors are using a subset of cases (not exposed) rather than neighborhood controls (without cancer), but more precise language is needed.

Response: We have now revised the text accordingly.

The analysis of pre-diagnosis exposure to the vaccine is a nice addition. Again, I encourage the use of “non-exposed cases” rather than “controls” for the sentence beginning on line 185.

Response: Thank the review for pointing out this. The text was revised accordingly.

The sentence on line 183 that begins with “However” focuses on the statistical significance, but in fact the point estimates of effect are nearly identical. That should be stated first,

followed by the observation that statistical significance was attenuated, and the attribution to small sample size is appropriate.

Response: The text was revised accordingly.

Figure 1. Y axis should be HR (in time not needed).

Response: It was revised accordingly.

Response #21 about anti-malarial drug concordance was not adequately addressed. It can be in at least two ways. A simple cross-tabulation of the exposures, or an analysis that considers the combination of the two exposures: no/no, yes/no, no/yes, yes/yes.

Response: We did a cross tabulation of the exposures listed in supplementary 4. We did not do the analyses as we used time-dependent Cox regression, and it was hard to define the exposure time for those with both vaccination and antimalarial medications.

The authors need to more strongly acknowledge that the findings are chance, perhaps due to residual confoounding, beyond the minimal comment that starts on line 303. For starters, line 144 should state that the sensitivity analyses were performed to "reduce" not "exclude" the possibility of chance findings.

Response: Thank the review for pointing out this. The text was revised accordingly.

Line 137 says that the proportional hazards assumption was tested, but the results are never given. They should be added.

Response: We showed the results in supplementary 5.

REVIEWERS' COMMENTS:

Reviewer #2 (Remarks to the Author):

All of the prior comments and suggestions have been fully addressed.

Reviewer #2 (Remarks to the Author):

All of the prior comments and suggestions have been fully addressed.

>>> We appreciate this positive response from the reviewer.
